# Individual signatures and environmental factors shape skin microbiota in healthy dogs

**DOI:** 10.1186/s40168-017-0355-6

**Published:** 2017-10-13

**Authors:** Anna Cuscó, Janelle M. Belanger, Liza Gershony, Alma Islas-Trejo, Kerinne Levy, Juan F. Medrano, Armand Sánchez, Anita M. Oberbauer, Olga Francino

**Affiliations:** 1 Molecular Genetics Veterinary Service (SVGM), Veterinary School, Universitat Autònoma de Barcelona, Barcelona, Spain; 2 Vetgenomics, Ed Eureka, Parc de Recerca UAB, Barcelona, Spain; 3 Department of Animal Science, University of California, Davis, CA USA; 4 Canine Companions for Independence, Santa Rosa, CA USA

**Keywords:** Skin, Canine, Microbiota, Microbiome, Dog, Season, Skin site, Pinna, 16S, Environment

## Abstract

**Background:**

The individual, together with its environment, has been reported as the main force driving composition and structure of skin microbiota in healthy dogs. Therefore, one of the major concerns when analyzing canine skin microbiota is the likely influence of the environment. Despite the dense fur covering, certain skin diseases exhibit differential prevalence among skin sites, dog breeds, and individuals.

**Results:**

We have characterized the normal variability of dog skin microbiota in a well-controlled cohort of a large number of Golden-Labrador Retriever crossed dogs (*N* = 35) with similar ages, related genetic background, and a shared environment. We found that the individual drives the skin microbiota composition and structure followed by the skin site. The main bacterial classes inhabiting dog skin in this cohort are Gammaproteobacteria and Bacilli. We also detected bacteria associated to the environment on different dog skin sites that could be reflecting the different degrees of exposure of each skin site and each dog. Network analyses elucidated bacterial interactions within and between skin sites, especially in the chin, abdomen, axilla, and perianal region, with the highly shared interactions probably representing an anatomical, behavioral, or environmental component. When analyzing each skin site independently to assess host-specific factors, we found that temporality (season of birth and time spent in the kennel) affected all the skin sites and specially the inner pinna. The most abundant taxon driving this difference was *Sphingomonas*. We also found taxonomic differences among male and female dogs on the abdomen, axilla, and back.

**Conclusions:**

We observed a large inter-individual variability and differences among skin sites. Host-specific variables, such as temporality or sex, were also shaping skin microbiota of healthy dogs, even in an environmental homogenous cohort.

**Electronic supplementary material:**

The online version of this article (10.1186/s40168-017-0355-6) contains supplementary material, which is available to authorized users.

## Background

Skin is a complex ecosystem inhabited by a high diversity of microorganisms, collectively referred to as the microbiota. These microbial communities not only inhabit, but also interact with the host cells impacting cellular function and immunity; likewise, the host immunity can influence the microbiota composition. This cross-talk between the host cells and the microorganisms maintains the homeostasis and the healthy status of an individual, and its disruption has been associated to disease [1–3].

The dense fur that covers almost all of a dog’s skin creates a homogenous microenvironment. However, some skin diseases show a preference for certain skin sites and for specific breeds [4]. Previous studies have described skin microbiota in healthy dogs [5–10], but only three of them included several skin sites to assess differences that may exist due to the anatomical location sampled [7, 9, 10]. Results from Rodrigues-Hoffmann and colleagues showed that haired skin regions presented higher diversity values than mucosal areas and mucocutaneous junctions [7], and a similar result was reported when comparing the inner pinna (hairy skin) to the perianal region (mucocutaneous junction) [9]. No differences among skin sites were detected when including microbiota samples from the dorsal neck, axilla, and abdomen [10].

Dog skin microbiota studies aimed at detecting differences between health and disease status have already been performed for canine atopic dermatitis [7, 8, 11]. Skin affected with atopic dermatitis in dogs presented a less diverse microbiota [7, 8] and increased proportions of *Staphylococcus* and *Corynebacterium* [8]. Moreover, dogs with allergen-induced atopic dermatitis presented higher proportions of *Staphylococcus* on the challenged site compared to the contralateral site [11].

In humans, skin microbiota differs among skin sites and among individuals [12]. On the one hand, the skin presents three main microhabitats depending on the physicochemical properties: sebaceous sites, mainly inhabited with *Propionibacterium* spp.; moist sites, with *Staphyloccocus* and *Corynebacterium* spp.; and dry sites, with gram-negative microorganisms [12, 13]. On the other hand, individual signatures of the skin microbiota are usually driven by low abundant species [14]. Following those first human studies describing skin microbiota, research then targeted key variables to ascertain if they drove skin microbiota structure and composition in the healthy individual. Variables assessed and found to have some effect on microbiota diversity, composition, and structure included those related to host such as sex [15–17], age [18–20] and racial origin [21–23] or related to environment such as birth delivery mode [24], hygiene [15, 23], cohabitation [6, 25], geography [22, 26, 27], and urbanization [20, 28, 29].

One of the major concerns when performing skin microbiota studies on dogs is the likely influence of the environment [30]. Our previous results suggest that the individual—together with its environment—was the main force driving skin microbiota composition and structure in a population of dogs from three different breeds and hair coats [9]. Rodrigues-Hoffmann and colleagues assessed some environmental variables, such as presence of fleas, time spent indoors vs outdoors, sex, or age, and did not detect significant associations between the microbiota and a particular environmental factor [7]. However, the dog cohort assessed was very variable and included 12 individuals from different breeds, ages, and households and likely obscured environmental effects. Two studies reported that dogs cohabiting together shared more skin microbiota [6, 10]. On the other hand, a recent longitudinal study using a cohort of 40 healthy dogs sampled in three skin sites assessed the effects of age, sex, breed, hair type, skin site, temporal point of collection, and cohabitation. They found that samples from different skin sites were more similar within the same dog and that microbiota structure was stratified by the temporal point of collection [10].

Skin microbiota has been suggested as a potential clinical tool in susceptibility, diagnosis, and treatment of dermatological diseases [31]. Characterizing the variability of skin microbiota in healthy dogs and determining which host and environmental variables are defining its structure and composition will extend the background to better design studies aimed to assess the altered skin microbiota in disease.

Here, we aimed to assess the variability of the canine skin microbiota in a homogeneous cohort of healthy dogs. We analyzed eight different skin sites in Golden-Labrador Retriever crossbred dogs (*N* = 35). The dogs were cohabiting together in the same kennel and sharing the same environmental conditions for at least 2.5 months. As most of the environmental variables were fixed, we also aimed to elucidate if any of the host factors were driving skin microbiota structure and composition in some skin sites. Finally, we compared the USA cohort with dogs from a European cohort.

## Methods

### Cohort description and dogs included

The USA cohort was composed of 35 Golden-Labrador Retriever crossed dogs, which were part of a larger service dog program. We sampled 20 females (14 yellow and 6 black) and 15 males (9 yellow and 6 black). Additional files 1 and 2 contain all the metadata associated with the dogs.

They were healthy companion dogs born in different households from breeding dogs that are also part of the program, where they were raised until 8 weeks of age at which time they were sent to individual puppy raisers until a minimum of 17 months of age. Dogs of similar ages then enter the kennel for training. In our cohort, 3 dogs born from January to February 2014 entered the kennel in August 2015, 13 dogs born from March to May 2014 entered in November 2015, and 19 dogs born from June to September 2014 entered in February 2016. The ages of the dogs at the time of sampling (April 2016) ranged from 19.5 to 27 months. Thus, these dogs had been living and playing together in a shared environment in the same kennel in Santa Rosa (California) for at least 2.5 months. Moreover, all dogs were fed a base diet from the same manufacturer (Eukanuba), with puppy and adult large breed diet fed at their different age stages. The water used for bathing, drinking, and cleaning the facilities comes from the municipal water system. The staff maintaining the kennel and feeding the animals were consistent the entire time the dogs were in the kennel.

Besides the shared environment, in most cases, the dogs had a shared genetic background: 33 out of 35 dogs sampled had at least one-half sibling or littermate in the study and only dogs 31, 19, and 14 were born from a unique set of progenitors (Additional file 1).

We analyzed the data obtained by “Individual” (35 dogs) and by “Site” (8 skin sites). We also analyzed the effect of host-specific variables. For each skin site, we grouped and analyzed the samples considering sex, coat color, and temporality. Temporality is a variable that we created to group dogs that were born in the same calendar season and that had spent a similar amount of time in the kennel. Thus, group T1 included 16 dogs born in winter-spring, from January to May (older), which have been living together in the kennel for at least 5.5 months (8.5 months for 3 of the dogs), and group T2 included 19 dogs born in summer from June to September (younger), which have been living in the kennel for 2.5 months at the time of sampling.

Dogs from the European cohort included 11 pets of different ages, households, and breeds (Beagle, French Bulldog, German Shepherd, and West Highland white terrier). They were all purebred dogs ranging from 3 months to 12 years of age. Nine of the dogs were described in a previous study [9], whereas two of them were sampled later (unpublished). These samples were processed in different batches along 18 months.

European and US samples were extracted in different years and in different facilities. One person was present in both DNA extraction procedures (AC). The samples were obtained with the same swabs, and the DNA was extracted with the same kit and protocol. PCRs were performed by the same person in the same facilities (AC), and sequencing was performed with the same equipment. To compare cohorts, all samples were analyzed together following the steps detailed below.

### Sample collection

**Fig. 1:**
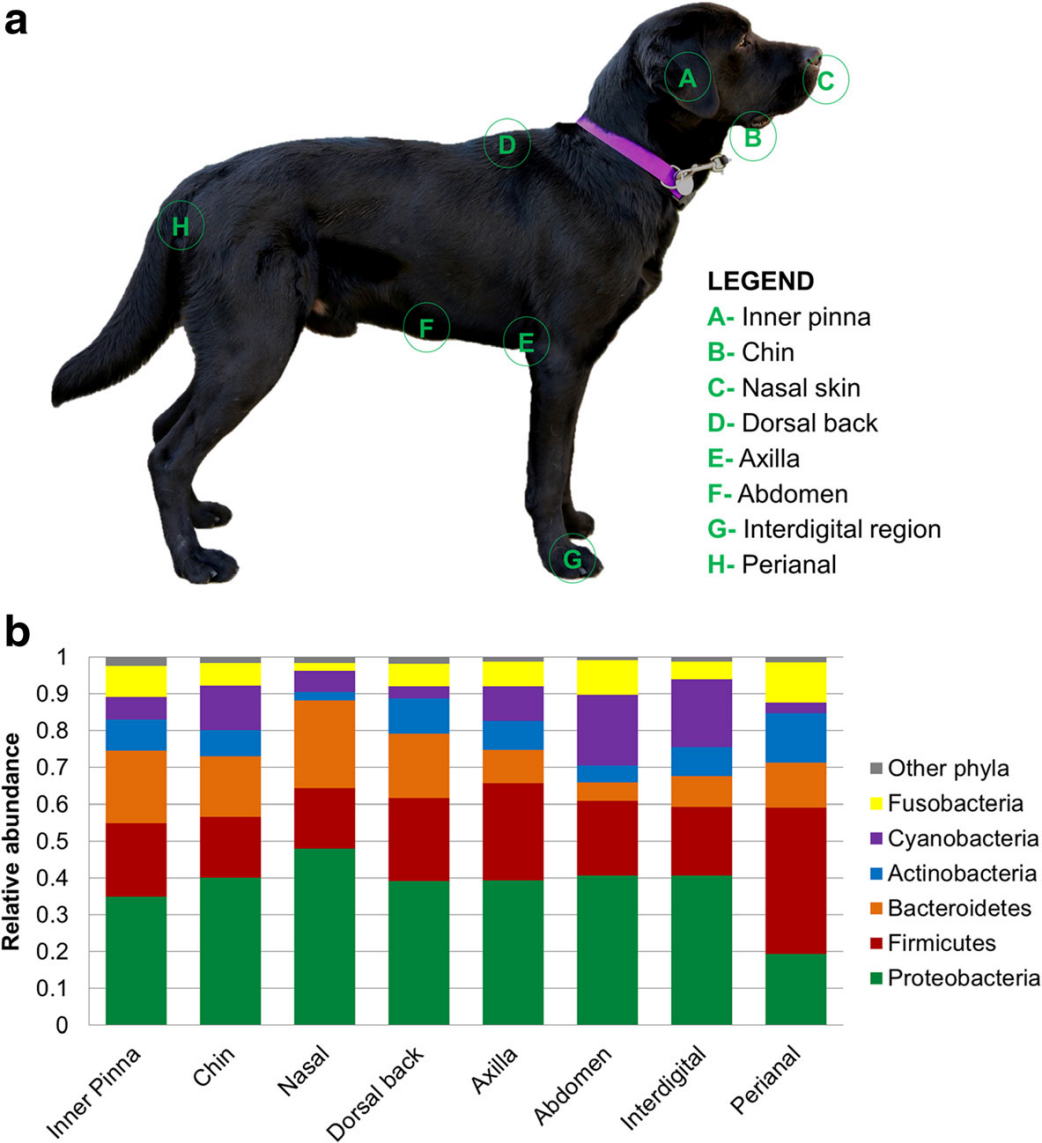
**a** Skin sites sampled and **b** taxonomic composition per skin site at phylum level

Skin microbiota samples were collected from eight regions taken from the right side of the dog: inner pinna, chin, nasal skin, back, axilla, abdomen, interdigital area, and perianal region. These regions are named as A, B, C, D, E, F, G, and H, respectively (Fig. 1a). Samples were obtained by firmly rubbing each area using Sterile Catch-All™ Sample Collection Swabs (Epicentre Biotechnologies, Madison, WI) soaked in sterile SCF-1 solution (50 mM Tris buffer (pH = 8), 1 mM EDTA, and 0.5% Tween-20). To minimize sample cross-contamination, the person sampling wore a fresh pair of sterile gloves for each individual. To minimize bias in sampling, only AO and AC sampled the dogs. The swabs were stored at 4 °C until DNA extraction, within the following 8 days (3 days, 2-day stop, 3 days).

### DNA extraction

Bacterial DNA was extracted from the swabs using the PowerSoil™ DNA isolation kit (MO BIO laboratories, Carlsbad, CA) under manufacturer’s conditions, with one modification. At the first lysis step, the swab tip with the sponge was cut and placed in the bead tube, until the first transference of the supernatant to a new tube. The remaining steps were performed as described by the manufacturer in exception of the elution step, which was performed on 50 μL of C6 instead of 100 μL to obtain a higher concentration. Samples from different skin sites and individuals were randomly extracted to avoid confounding a batch effect with an actual effect. DNA extractions were performed within the following 8 days in random batches of samples to avoid confounding technical biases with actual ones. DNA samples (50 μl) were stored at − 20 °C until further processing. To assess for contamination from the laboratory or reagents, two blank samples were processed: one with a sterile swab tip and the other without the sterile swab tip.

### PCR amplification and massive sequencing

V1–V2 regions of 16S rRNA gene were amplified using the widely used primer pair F27 (5′-AGAGTTTGATCCTGGCTCAG-3′) and R338 (5′-TGCTGCCTCCCGTAGGAGT-3′), which targets 311 bp in *E. coli* genome. We choose V1–V2 hypervariable regions because they had been suggested to be a better choice for human skin microbiota among others [32]. PCR mixture (25 μl) contained 2 μl of DNA template, 5 μl of 5× Phusion® High Fidelity Buffer, 2.5 μl of dNTPs (2 mM), 0.2 μM of each primer, and 0.5 U of Phusion® Hot Start II Taq Polymerase (Thermo Scientific, Vilnius, Lithuania).

The PCR thermal profile consisted of an initial denaturation of 30 s at 98 °C, followed by 30 cycles of 15 s at 98 °C, 15 s at 55 °C, 20 s at 72 °C, and a final step of 7 min at 72 °C. Samples that did not amplify the first time were repeated increasing cycles to 33. To assess possible reagent contamination, each PCR reaction included a no template control (NTC) sample.

For each amplicon, quality and quantity were assessed using Agilent Bioanalyzer 2100 (Agilent, Santa Clara, CA) and Qubit™ fluorometer (Life Technologies, Carlsbad, CA), respectively. Both primers included sequencing adaptors at the 5′ end and forward primers were tagged with different barcodes to pool samples in the same sequencing reaction, which results in a 415 bp fragment.

Each sequencing pool included 40 barcoded samples that were sequenced on an Ion Torrent Personal Genome Machine (PGM) with the Ion 318 Chip Kit v2 and the Ion PGM™ Sequencing 400 Kit (Life Technologies, Carlsbad, CA) under manufacturer’s conditions.

### Quality control of the sequences and OTU picking

Raw sequencing reads were demultiplexed and quality-filtered using QIIME 1.9.1 [33]. Reads included presented a length greater than 300 bp, a mean quality score above 25 in sliding window of 50 nucleotides, no mismatches on the primer, and default values for other quality parameters. After that, quality-filtered reads were processed using vsearch v1.1 pipeline [34]: a first de-replication step was applied, followed by clustering into operational taxonomic units (OTUs) at 97% similarity with a de novo approach and finally chimera checking was performed using UCHIME [35] de novo. The raw OTU table was transferred into QIIME 1.9.1, and taxonomic assignment of representative OTUs was performed using the Ribosomal Database Project (RDP) Classifier [36] against Greengenes v13.8 database [37]. Alignment of sequences was performed using PyNast [38]. We sequentially applied extra filtering steps in aligned and taxonomy-assigned OTU table to filter out (1) sequences that belonged to chloroplast class, (2) sequences representing less than 0.005% of total OTUs (as previously done in [39]), (3) sequences that belonged to *Shewanellaceae* and *Halomonadaceae* families, which were highly represented in the NTC of the repetition chip (performed with an increased cycle number) and considered contamination from the reagents.

Samples 17G and 27A did not amplify and they could not be sequenced. We performed downstream analysis at a depth of 11,000 sequences per sample: 1D, 30C, 6G, and 8G failed this parameter and were discarded for posterior analyses. Also, NTC and Blank with a swab tip presented some amplification but failed to reach 11,000 sequences per sample; blank without the swab tip could not amplify.

### Downstream bioinformatics analyses

Downstream analyses were performed using QIIME 1.9.1 [33] with the filtered OTU table. To standardize samples with unequal sequencing depths, analyses were performed using random subsets of 11,000 sequences per sample.

Alpha diversity analysis assesses the diversity within a sample. Two different metrics were used for the alpha diversity: observed species to assess richness and Shannon index to assess evenness. Data were tested for normality by the Shapiro-Wilk test implemented in *R*. As the values were not following a normal distribution, we assessed statistical significant differences in alpha diversity values among groups with 999 permutations using the non-parametric Monte Carlo permutation test and corrected the *p* value through false discovery rate.

Beta diversity analysis assesses the similarities among samples of the same community. Beta diversity was performed using both weighted and unweighted UniFrac distance metrics [40]. Weighted UniFrac considers phylogeny, taxa, and relative abundances, whereas unweighted UniFrac only considers phylogeny and taxa. Those distance matrices were used to create PCoA plots. ANOSIM and adonis statistical methods were applied to evaluate the extent of a variable effect on the dissimilarity of microbial communities.

Linear discriminant analysis (LDA) effect size (LEfSe) [41] was used to compare groups and to identify taxa whose abundance is differentially abundant between groups (*α* = 0.05 and with an LDA score > 3.0).

CoNet [42], which is implemented as an application in Cytoscape [43], was applied to infer networks among skin sites using bacterial families that presented a median relative abundance higher than 0.05% in each specific site. In CoNet, we used five different algorithms (Pearson’s correlation, Spearman’s correlation, Kullback-Leibler dissimilarity distances, Bray-Curtis dissimilarity distances and mutual information similarity) since the combination of their results allows the appropriateness of scoring measures to sparse count data and determination of statistical significance, as stated by the authors [44]. The results of the five methods were merged using Simes *p* value. We performed a first permutation step, followed by a bootstrap analysis corrected for false discovery rate (*α* = 0.05).

## Results

We analyzed the variability of the canine skin microbiota in eight different skin sites from a healthy homogenous and well-controlled cohort of Golden-Labrador Retriever crossbred dogs cohabiting together in the same kennel in the USA (*N* = 35) (see Additional files 1 and 2 for the associated metadata). At the time of sampling, dogs ranged in age from 19.5 to 27 months old and had been living and playing together in a shared environment for at least 2.5 months. All dogs were fed a base diet from the same manufacturer in their different age stages and shared the municipal water used for bathing, drinking, and cleaning the facilities. The staff maintaining the kennel and feeding the animals were consistent over the entire stay of these dogs in the kennel.

We sampled microbiota from eight skin sites: inner pinna, chin, nasal skin, dorsal back, axilla, abdomen, interdigital region, and perianal area, which are respectively named as A, B, C, D, E, F, G, and H (Fig. 1a). These anatomic sites were selected to represent the regional diversity of the canine skin [4]. Samples 17G, 27A, 1D, 30C, 6G, and 8G failed at some processing point and were discarded for posterior analyses (see the “Methods” section for more detail).

### Individual and skin sites: taxonomy and diversity analysis

We found a total of 2216 bacterial OTUs living on dog skin *(*Additional file 3 *)* that were taxonomically classified into 17 phyla, 41 classes, 62 orders, 128 families, and 242 genera. Specifically, the main phyla inhabiting dog skin of healthy dogs were Proteobacteria, Firmicutes, Bacteroidetes, Actinobacteria, Cyanobacteria, and Fusobacteria followed by TM7, Tenericutes, and others with lower abundances (Fig. 1b).

Proteobacteria was usually the main phylum found on the skin of our cohort (Fig. 1b and Additional file 4). Fusobacteria were most frequently found in the perianal regions; however, when Fusobacteria colonized the haired skin, the distribution was individual-specific. That is, there were a few dogs with high abundance of Fusobacteria in several regions whereas other dogs had almost no Fusobacteria. Within the Fusobacteria enriched individuals, usually the highest percentages were found in the abdomen samples. Finally, Cyanobacteria phylum was mainly present with high abundances in the abdomen, interdigital region, and the chin of specific individuals (Additional file 4).

Grouping the samples per individual significantly explained 23% and 22% of the variation in unweighted and weighted UniFrac distance matrices (Table 1), suggesting that the main force driving the variability of skin microbiota in our samples was the individual. When assessing alpha diversity, no statistical significant differences were observed among individuals (Additional file 5A), probably due to the large differences in alpha diversity values within the same dog. Thus, some dogs that could seem less diverse because most of the skin sites presented less diversity usually presented average values in the inner pinna or perianal region, giving no statistical significant differences among individuals.

**Table 1:** Clustering of the samples per biological and technical variables

	Unweighted UniFrac Adonis *R* ^2^	Weighted UniFrac Adonis *R* ^2^
Individual	0.23**	0.22**
Skin site	0.12**	0.17**
Storage time	0.05**	0.05**
Chip	0.03*	–
Person extracting	0.02*	0.02*
Sampler	0.01*	0.01*

*−* no significant clustering

**p value = 0.001; *p value < 0.05

On the other hand, clustering samples per skin site explained 12% and 17% of the variation in unweighted and weighted UniFrac distance matrices. Differences in microbiota structure were also significant among almost all pairs of skin sites, with the exception of the interdigital region when compared to the abdomen or axilla. We found the greatest differences when comparing any skin site to the perianal region followed by the nasal skin (Additional file 6). Differences in alpha diversity among skin sites were prevalent. The inner pinna displayed the greatest diversity when compared to all the other sites and was statistically different to all (*p* value = 0.028) but the chin site. The chin, when considering observed species, was significantly more diverse than the axilla (*p* value = 0.028) (Additional file 5B).

Focusing on taxonomic analyses, we found that bacteria from the Gammaproteobacteria class were the most abundant in dog skin microbiota, with the exception of perianal regions where Bacilli class from Firmicutes phylum were the most abundant.

**Fig. 2:**
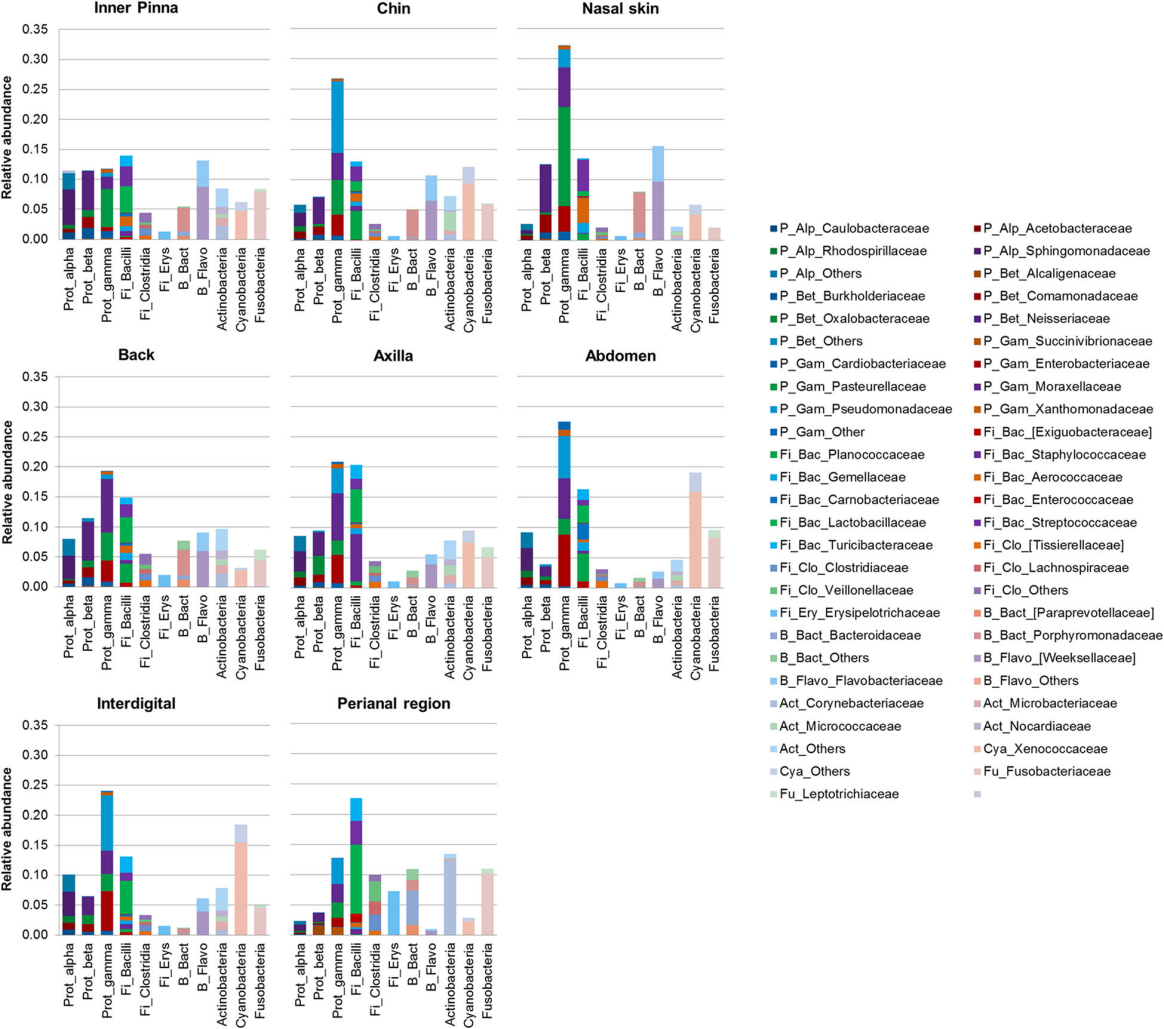
Taxonomic profiles per skin site. Taxa summary bar plots per class colored by main families within each skin site. Prot_alpha: Proteobacteria_Alphaproteobacteria; Prot_beta: Proteobacteria_Betaproteobacteria; Prot_gamma: Proteobacteria_Gammaproteobacteria; Fi_: Firmicutes; Fi_Erys: Firmicutes_Eryspelotrichi; B_Bact: Bacteroidetes_Bacteroidia; and B_Flavo: Bacteroidetes_Flavobacteriia

Skin sites shared most of the taxa, but presented also specific taxonomic patterns: the abundance and distribution varied significantly among skin sites, and unique taxa were identified in some of the sites. Figure 2 shows different bar plots, colored by the main families found in the skin. The families that were differentially distributed (LDA score > 3, *p* value = 0.05) are shown in Additional file 7.

The inner pinna had a higher amount of Proteobacteria phylum when compared to other skin sites, with Gammaproteobacteria, Alphaproteobacteria, and Betaproteobacteria classes being the main representatives. Bacilli (Firmicutes) and Flavobacteriia (Bacteroidetes) were present in similar abundances to Proteobacteria. Moreover, inner pinna presented many different and less abundant bacteria.

The chin region was enriched in Gammaproteobacteria, with Pseudomonadaceae as the main representative family. The nasal skin was also enriched in Gammaproteobacteria, but the main representative family was Pasteurellaceae. Both families were differentially distributed in their respective skin site.

The back and axilla had quite similar taxonomic patterns: the main bacterial class was Gammaproteobacteria, with Moraxellaceae as the main family, followed by Bacilli, with Lactobacillaceae as one of the main families. The greatest taxonomic difference between both sites was the higher abundance of Staphylococcaceae (Bacilli class) in the axilla, which was also differentially distributed when compared to the other skin sites.

The abdomen and interdigital regions had similar taxonomic patterns, where most of the bacteria were Gammaproteobacteria, specifically from Enterobacteriaceae, Moraxellaceae, and Pseudomonadaceae families, followed by Cyanobacteria, specifically Xenococcaceae family. However, Planococcaceae was found in the abdomen but not in the interdigital region.

Finally, the perianal region was the skin site that presented the most differentiated pattern in dog skin microbiota. The main phylum was Firmicutes, especially Bacilli, followed by Actinobacteria. Many different families from different phyla were differentially distributed in the perianal region, indicating that it was the most divergent skin site (Additional file 7). Most of the abundant families in the perianal region were also differentially distributed when compared to the other skin sites. Some of them were Erysipelotrichaceae, Lachnospiraceae, Lactobacillaceae, and Veillonellaceae (Firmicutes); Corynebacteriaceae (Actinobacteria); and Bacteroidaceae (Bacteroidetes). The perianal region was also enriched in Fusobateriaceae, despite not being statistically differentially distributed when compared to other skin sites.

### Skin sites: network analysis

**Fig. 3:**
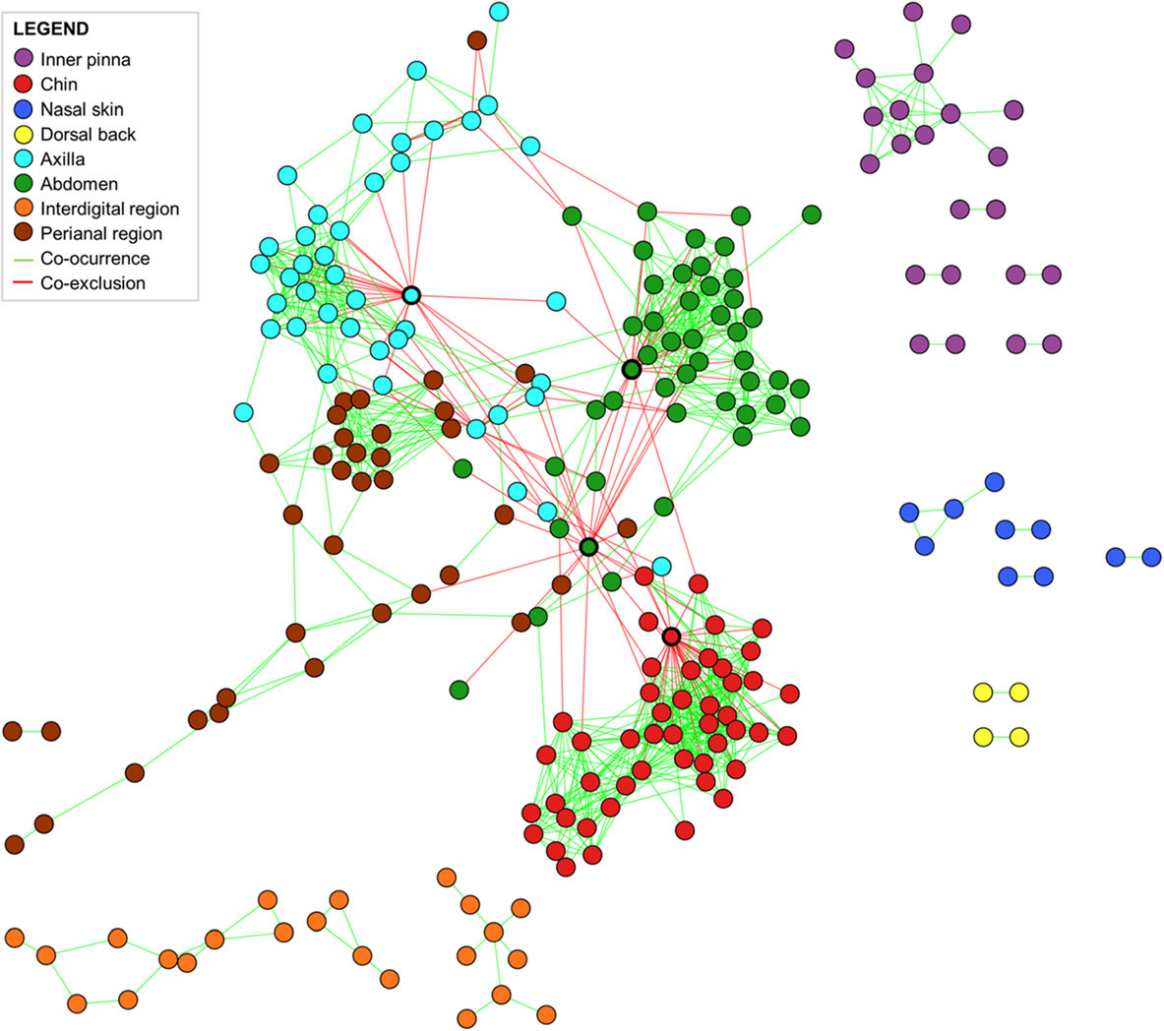
Significant co-occurrence and co-exclusion interactions among the abundant families (> 0.005%) in the dog skin microbiota. Nodes are colored depending on the skin site they are found; nodes with a wider black circle are those highly connected mutual exclusion nodes; edges are green to represent co-occurrence patters and red to represent co-exclusions. Data associated with the complete network can be found in Additional file 8

A network analysis detects bacterial relationships, within and among different ecological niches. The global network for all the skin sites considering the most abundant families allowed us to understand more deeply skin microbiota relationships in our cohort (Fig. 3, Table 2, and Additional file 8). Some bacterial species interacted specifically in the same skin site, whereas other bacterial species interacted among different skin sites. Thus, we have different ecological niches within the skin.

**Table 2:** Summary statistics of microbial interactions in the skin of a cohort of healthy dogs

	Chin	Abdomen	Axilla	Perianal region	Inner pinna	Nasal skin	ID area	Dorsal back
Total interactions	373	226	179	93	35	7	23	2
Common interaction	139	103	104	43	13	4	13	2
Unique interactions	234	123	75	50	22	3	10	0
Inter-site interaction^a^	3	20	10	12	0	0	0	0
% of unique interactions	63%	54%	42%	54%	63%	43%	43%	0%
% of co-occurrence	92%	88%	79%	100%	100%	100%	100%	100%

*ID* interdigital

^a^Inter-site interactions represent families from a specific skin site, affecting other families from another skin site

The chin, abdomen, axilla, and perianal region had the highest number of interactions, with 373, 226, 179, and 93, respectively, and also some extra interactions among families of other skin sites (Table 2 and Additional file 8). On the other hand, the inner pinna, nasal skin, interdigital region, and dorsal back presented a lower number of interactions and no inter-site interactions, as shown in Fig. 3. The inner pinna had 35 family interactions, interdigital region 23, nasal skin 7, and dorsal back 2.

In some cases, specific taxonomic interactions were found within different skin sites. We identified six co-occurrence interactions highly spread among different skin sites (present in 4 out of 8 skin sites): Neisseriaceae and Weeksellaceae; Neisseriaceae and Xenococcaceae (in the chin, axilla, abdomen, and perianal); Sphingomonadaceae and Caulobacteraceae (in the inner pinna, axilla, abdomen, and perianal); Sphingomonadaceae and Nocardioidaceae (in the inner pinna, axilla, abdomen, and interdigital region); Sphingomonadaceae and Oxalobacteraceae (in the inner pinna, chin, abdomen, and interdigital); and Weeksellaceae and Flavobacteriaceae (in the chin, axilla, abdomen, and interdigital). However, most interactions (517 out of 703) were exclusive from one specific skin site (Additional file 8).

This global network demonstrated that most interactions in the canine skin were co-occurrence relationships rather than mutual exclusion. Among mutual exclusion interactions, few nodes were negatively linked to many different families within a skin site (circles marked with a wider black line in Fig. 3), showing an apparent invasive pattern. That was seen for Pseudomonadaceae family in the axilla, chin, and abdomen and also for Enterobacteriaceae family in the abdomen. When blasting the most abundant OTUs from the highly connected mutual exclusion nodes, we found that the main genera were *Pseudomonas* (for Pseudomonadaceae) and *Erwinia* and *Pantoea* (for Enterobacteriaceae) (Additional file 9).

### Effect of host-specific and technical variables on canine skin microbiota

In order to assess if any host-specific variable defined the skin microbiota composition or structure in any of the skin sites, we inspected the alpha and beta diversity of each skin site grouped by the different host-specific variables such as sex, coat color, temporality, or recent surgery and assessed statistical significance except for the recent surgery due to the small sample size. Temporality was a variable that classified all of the animals within two groups: T1 includes those dogs born from January to May, which have been in the kennel at least 5.5 months, whereas T2 includes those dogs born from June to September, which have been in the kennel 2.5 months (detailed explanation is in the “Methods” section).

**Fig. 4:**
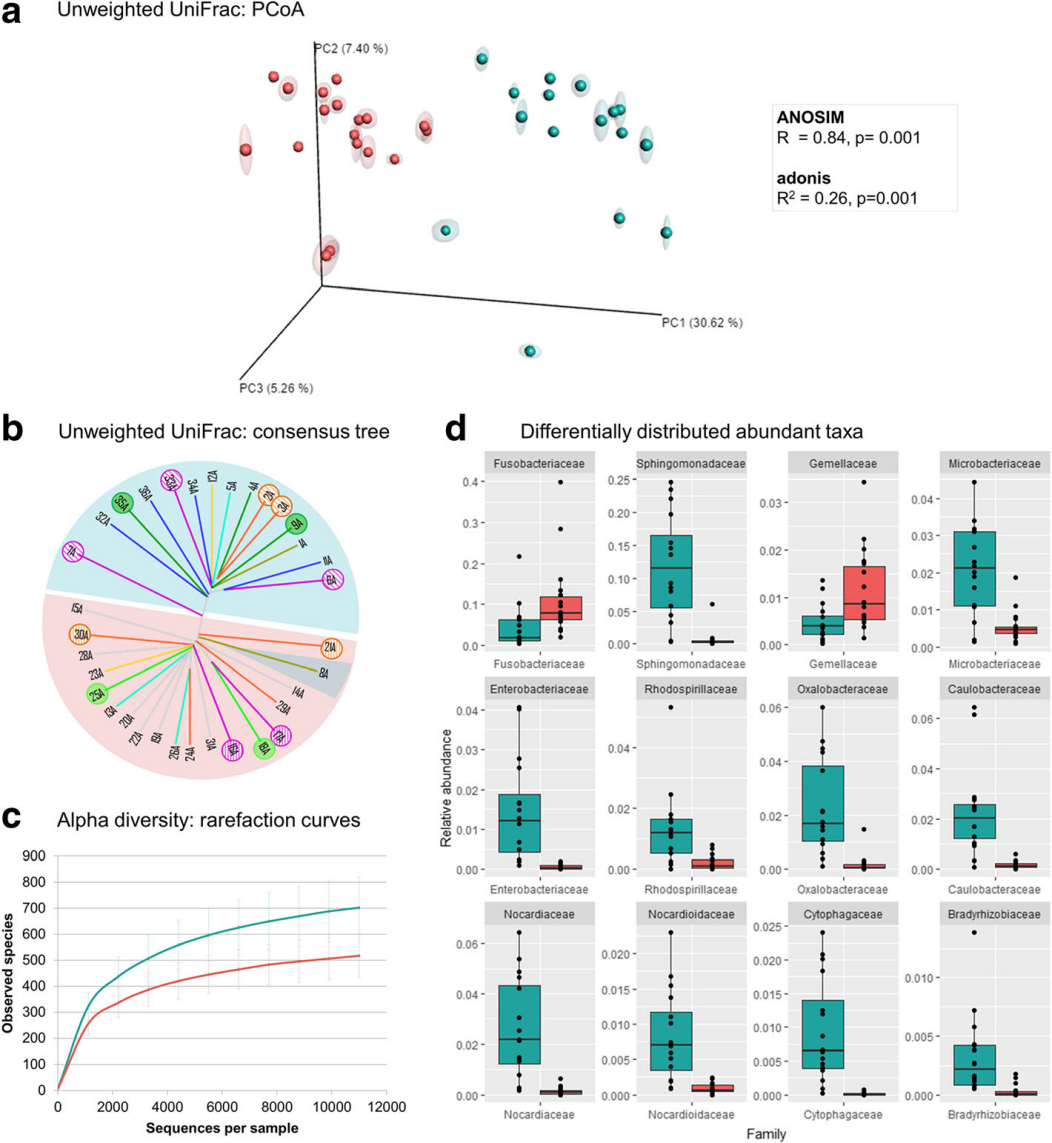
Effect of temporality on the inner pinna. Color blue represents T1 group (dogs born from January to May that had been in the kennel for at least 5.5 months) and color red represents T2 group (dogs born from June to September that had been in the kennel for 2.5 months). **a** Unweighted UniFrac PCoA beta diversity plot. **b** Unweighted UniFrac consensus tree: dogs sharing sire present same-colored branches and littermates are circled and colored with a common pattern within a group. **c** Alpha diversity rarefaction curves using observed species metrics. **d** Boxplots of the main differentially distributed families: those include families with abundances > 1% in any group and also LEfSe significant (LDA score > 3.0, *p* value < 0.05)

Temporality was the variable that explained ubiquitously a significant amount of variation for all the skin sites. Temporality significantly affected the microbiota composition (unweighted UniFrac) and also the community structure (weighted UniFrac) in all skin sites (Table 3; Additional file 10). This effect was especially large on the inner pinna, almost coincident with PC1 component, explaining 26% of the variation among samples and with an ANOSIM *R* value of 0.84, suggesting great dissimilarity between T1 and T2 (Table 3, Fig. 4a). In the other skin sites, temporality explained more than 9% of the variation (except for the nasal skin), with ANOSIM *R* values ranging from 0.24 to 0.38 for the different sites.

**Table 3:** Host-specific variables that cluster samples in specific skin sites

		Unweighted UniFrac		Weighted UniFrac	
Skin site	Variable	ANOSIM *R*	Adonis *R* ^2^	ANOSIM *R*	Adonis *R* ^2^
Inner pinna (A)	Temporality	0.84**	0.26**	0.41**	0.22**
Axilla (E)	Temporality	0.38**	0.11**	0.09*	0.07*
Dorsal back (D)	Temporality	0.37**	0.13**	0.28**	0.14**
Interdigital (G)	Temporality	0.28**	0.11**	0.09*	0.07*
Abdomen (F)	Temporality	0.28**	0.10**	0.09*	0.07*
Perianal (H)	Temporality	0.27**	0.09**	–	–
Chin (B)	Temporality	0.24*	0.10*	0.10*	0.08*
Abdomen	Sex	0.13*	0.05*	0.24*	0.11**
Nasal skin (C)	Temporality	0.11*	0.05*	0.06*	–
Back	Sex	–	0.05*	–	–
Axilla	Sex	–	–	–	0.06*

Statistical significance of the clustering calculated through ANOSIM and Adonis values for beta diversity unweighted and weighted UniFrac matrices

– no significant clustering

**p value = 0.001, *0.05 > *p* value > 0.001

Delving into the effect of this variable on the inner pinna skin microbiota, we visually corroborated the pattern in the unweighted UniFrac consensus tree (Fig. 4b): two clear clusters were elucidated matching with T1 and T2 groups (except dog 8). Even when looking at the genetic background, we could see that littermates were usually as similar as any other dog in the same group (except dogs 2 and 3) and sharing the sire did not make dogs resemble more in skin microbiota. Moreover, dogs from the T1 group were significantly more diverse than those from the T2 group (Fig. 4c). Finally, LEFSe analysis detected 61 families differentially distributed in the inner pinna when clustering in these two groups (Additional file 11) and those with higher relative abundances are plotted in Fig. 4d. The most representative taxa differentially distributed in T1 and T2 are Sphingomonadaceae, Microbacteriaceae, Oxalobacteraceae, Caulobacteraceae, Nocardiaceae, and others with lower abundances. Sphingomonadaceae provides the greatest difference: it is highly present in the inner pinna of T1 dogs (with a median value around 11% of total microbiota composition), whereas it is almost absent on T2 dogs.

The sex of the dog also explained some variation. The microbiota community structure in the abdomen was better explained by the variable sex (11% of the variation in the weighted UniFrac plot) rather than temporality. This variable also explained to a lesser extent some variability of the microbiota composition (unweighted UniFrac) in the dorsal back and the community structure (weighted UniFrac) in the axilla (Table 3). Considering the three skin sites affected by sex (abdomen, back, and axilla), we could see that males had an overrepresentation of bacteria from Fusobacteria phylum, with *Sneathia* and *Fusobacterium* genera; other genera such as *Actinomycetospora*, *Gemella*, *Parvimonas*, *Brevundimonas*, and phylum SR1 were also overrepresented on males. Females had an overrepresentation of Enterobacteriaceae family (Table 4).

**Table 4:** Differentially abundant taxa associated to sex

		Abdomen		Axilla		Back	
Phylum	Family or genus	Female	Male	Female	Male	Female	Male
Fusobacteria	Fusobacteriales (order)	1.70%	21.45%	1.64%	13.44%	3.54%	9.55%
Fusobacteria	Leptotrichiaceae	0.23%	2.70%	0.24%	3.53%	0.82%	3.00%
Fusobacteria	*Sneathia*	0.01%	0.34%	0.05%	0.25%	0.21%	0.47%
Fusobacteria	*Fusobacterium*	NS	NS	1.41%	9.91%	2.72%	6.54%
Actinobacteria	*Actinomycetospora*	NS	NS	0.00%	0.04%	0.00%	0.15%
Firmicutes	*Gemella*	0.19%	3.04%	0.51%	1.61%	NS	NS
Firmicutes	*Parvimonas*	NS	NS	0.16%	1.82%	0.55%	1.15%
Proteobacteria	*Brevundimonas*	NS	NS	0.00%	0.01%	0.00%	0.01%
SR1	SR1	NS	NS	0.05%	0.19%	0.14%	0.44%
Proteobacteria	Enterobacteriaceae	14.08%	1.31%	7.78%	0.69%	NS	NS

Relative abundances of main taxa found to be differentially distributed (LDA score > 3, *p* value < 0.05) between males and females in at least two out of the three skin sites affected

*NS* no significant differences

We delved deeper into the five dogs that had undergone surgery followed by a medical treatment prior to sampling (Table 5 and Additional file 5C). Dogs 14, 15, 16, and 17 presented reduced alpha diversity values in several skin sites, being the chin and abdomen always affected, whereas alpha diversity values of the inner pinna, nasal skin, and back were not reduced in any dog. Dog 20, which underwent surgery 3 months before sampling, presented average alpha diversity values.

**Table 5:** Information of the dogs that had undergone surgery prior to sampling

Individual	Surgery date	Surgery type	Medicines	From	To	Sites w. reduced α-diversity ^a^
Dog 14	2016/04/08	Spay	Amoxicillin (antibiotic) + Previcox (anti-inflammatory)	2016/04/08	2016/04/13	Chin and abdomen
Dog 15	2016/04/18	Spay	Amoxicillin (antibiotic) + Previcox (anti-inflammatory)	2016/04/18	2016/04/23	Chin, axilla, abdomen, and ID region
Dog 16	2016/03/30	GI obstruction	Pepcid AC (antihistamine) + Tramadol (analgesic)	2016/04/01	2016/04/06	Chin, axilla, abdomen, ID region, and perianal area
Dog 17	2016/04/12	Spay	Previcox (anti-inflammatory)	2016/04/12	2016/04/16	Chin, axilla, abdomen, and perianal
Dog 20	2016/01/05	Spay	Rimadyl (anti-inflammatory)	2016/01/05	2016/01/10	None

^a^Reduced alpha diversity values include those ones that are half or less than the median alpha diversity of that specific skin site of the non-surgery dogs (Additional file 4C)

The coat color was not significantly explaining the skin microbiota structure or composition in any skin site.

We performed an additional analysis comparing the US cohort with some dogs from a European cohort (see the “Methods” section for more details). The European cohort included dogs from different breeds, ages, and inhabiting in different households that had been previously processed along 18 months in different batches. Two clear clusters were observed: dogs from the European cohort were grouping in a tight cluster, whereas the cluster for the US dogs was more diffuse. The grouping of back samples was stronger than the grouping of abdomen samples (ANOSIM *R* = + 0.68), and it significantly explained 12% of the variation in unweighted UniFrac (Additional file 12).

We also assessed the effect of other technical variables, such as sampler, person extracting, chip, and storage time. These technical variables explained 5% or less of the variation in the PCoA plots (Table 1).

## Discussion

Our results suggest that the main force driving the skin microbiota composition is the individual, followed by the skin site, even in a homogeneous cohort of dogs cohabiting and interacting together. This is in line with what we found previously in a cohort of nine healthy dogs from three different breeds, although in that study we could not elucidate whether the individual effect was real or represented an environmental influence [9]. Here, we homogenized the cohort to account for different effects: same crossbreed dogs, same age, same diet, and same environment. An individual effect had also been reported as the main driver of fungal skin microbiota structure and composition in dogs from heterogeneous cohorts [45] and had been suggested to also affect bacterial skin microbiota in dogs, despite the fact that the individual was not assessed directly [7]. Similarly, these two factors also shaped human skin microbiota, with great variability within several skin sites of an individual and between individuals having been reported [12, 46, 47].

The human skin has three main microhabitats (moist, dry, and sebaceous) inhabited by a specific taxa [13, 48]. Although the three microhabitats clearly identified in humans were not seen in dogs [30], Rodrigues-Hoffmann and colleagues reported significant differences between haired and mucosal or mucocutaneous junctions [7], which coincide with our current observation. Here, we found that the mucocutaneous perianal region and, to a lower extent, nasal skin presented different community structures (weighted UniFrac) as well as lower alpha diversity values when compared to all other haired skin regions.

Globally, in our cohort, Gammaproteobacteria followed by Bacilli were the most abundant classes in all regions in exception of perianal region with the same classes but the opposite order. A previous study including the dorsal neck, abdomen, and axilla samples from 40 domestic dogs inhabiting different households found Gammaproteobacteria and Bacilli as main classes, but also Actinobacteria [10]. On the other hand, Hoffmann and colleagues [7] detected different abundant classes depending on the skin site: Betaproteobacteria was the most common in the concave pinna, dorsal lumbar, and ear; Actinobacteria, in the axilla and interdigital skin; Gammaproteobacteria, in the nostril; and Clostridia and Bacteroidia, in the perianal region. Finally, in our previous study, we found Bacilli as the main class for all the skin sites with the exception of inner pinna that had Alphaproteobacteria [9]. Thus, as the inter-individual variability is large, independent studies led to similar results only when a large number of individuals are included.

Network analysis elucidated the overall community organization throughout the skin of our canine cohort, with more than 40% of the interactions exclusive of each site, demonstrating a skin site signature. The back skin presented only two interactions, and both of them were back-exclusive, and probably, other interactions remain hidden because only abundant families were included for network analysis. Among the rest of the skin sites, the inner pinna and chin were the sites that presented a higher proportion of unique interactions, suggesting stronger specialization or influences. On the one hand, the inner pinna is an anatomically and environmentally isolated site when compared to other skin sites. On the other hand, we suggest that the chin presented influences of both drinking water and oral microbiota. The most abundant families were Xenococcaceae and Pseudomonadaceae, which had been isolated in several water sources [49, 50]. Moreover, the following abundant families, such as Fusobacteriaceae, Moraxellaceae, Porphyromonadaceae, Neisseriaceae, and Flavobacteriaceae, were previously found as main taxa in canine oral microbiota [8, 51].

Network analyses also detected a high number of mutual exclusions when Enterobacteriaceae were abundant in the abdomen or Pseudomonadaceae were abundant in the abdomen, axilla, or chin. When blasting the OTUs that presented this apparently invasive pattern (Additional file 8), we found that the ones belonging to Enterobacteriaceae family had been mainly isolated from soil or plant surfaces [52, 53], whereas those from Pseudomonadaceae family had been mainly isolated from soil and different sources of water [50]. Thus, we suggest that this pattern is representing a recent exposure to the environment prior to sampling of some of the dogs.

Other bacteria with a likely environmental origin are Xenococcaceae with *Chroococcidiopsis* as its main genus. Bacteria from this genus had been mainly isolated from freshwater environments including lakes, soil, or inside of rocks [49]. Moreover, they have already been detected in healthy dog skin [7, 9]. The presence of these bacteria with high abundance at the interdigital and abdominal regions may suggest these two regions are more susceptible to environmental influences, which seems reasonable since these two skin sites have direct contact with the ground.

The skin sites could be classified based upon two patterns. The first pattern included sites having a high number of interactions among abundant families, with some interactions with other skin sites (chin, axilla, abdomen, and perianal region). The second pattern included sites having a lower number of interactions and displayed exclusively within-site interactions (pinna, nasal skin, dorsal back, and interdigital area). We suggest that the inter-site relationships could be related to topographical, behavioral, and environmental factors. The chin is juxtaposed to the mouth, which is a main entrance for the environment through licking, eating, or drinking water. Dogs could lap the same water in which they are playing, and they usually lick themselves, which could explain some interactions among those sites. Additionally, the abdomen and axilla are anatomically continuous on the ventral side of the dog and close to the ground facilitating interactions with the environment and between the two skin sites. Furthermore, dogs may come into contact with fecal matter, which could be the origin of shared OTUs among the abdomen, axilla and perianal regions. The main families of the second pattern, constituted by the inner pinna, dorsal back, interdigital area, and nasal skin, were only interacting with other families in the same skin site, suggesting that both anatomical isolation and stronger effects of other microbiota (nostril microbiota, for nasal skin, and soil microbiota, for interdigital region) may account for the exclusive within-site interactions.

With this general overview, we sought to elucidate if any host-specific variable determined the observed diversity, composition, and/or community structure in any of the skin sites. When considering the temporality, the two groups were significantly different: T1, which includes dogs born from January to May that had spent at least 5.5 months in the kennel, and T2, which includes dogs born from June to September that had spent 2.5 months in the kennel. This effect was highest on the inner pinna, with a significant ANOSIM *R* value of + 0.84, suggesting great dissimilarity between groups associated to temporality (Fig. 4). The main taxonomic difference among the inner pinna from both groups was due to Sphingomonadaceae, specifically *Sphingomonas*. These taxa are classically considered air- and dust-borne [54], although they have also been identified on dog skin microbiota [6, 9] and in animal sheds [54, 55], even specifically on dogs’ [56]. These bacteria are cultivable at temperatures ranging from 4 to 28  °C, but not at 37 °C [54]. Independent studies of grapevine microbiome showed a link between the abundance of Sphingomonadaceae and lower temperatures [57, 58]. The bacterial pool of the environment and the air is constantly shaped with seasonal characteristics such as humidity, UV light, and temperature [59], and it could be shaping to some extent the skin microbiota of dogs via environmental selection. We cannot distinguish if the effect was correlated to season of birth or time spent in the kennel, since the older dogs were born in colder seasons, which could have an effect in the initial colonization, but these dogs also entered the kennel in autumn and spent more cold months at the kennel.

Although it is difficult to elucidate which bacteria are really microbiota and which are only transient members from the environment, in our case, we sampled at a unique time point and still found significant differences regarding temporality. Therefore, Sphingomonadaceae and some of the other taxa differentially distributed (Fig. 4) would potentially be considered as normal colonizers of dog skin microbiota. An analogous example would be the genus *Enhydrobacter* that was commonly found in air and surfaces of the built environment of Hong Kong [60] and also presented high abundances in the skin of Chinese individuals [22, 61]. Also, Amerindian individuals, who spend more time outdoors than westernized individuals, presented a very diverse skin microbiota with a high proportion of bacteria commonly regarded as environmental [26].

Besides temporality, sex had a significant effect on the abdomen, back, and axilla microbiota of our cohorts. Female dogs presented an overrepresentation of Enterobacteriales and Enterobacteriaceae families, coinciding with what was previously reported on the hands of humans [15].

Dogs that had undergone surgery within the previous month presented low alpha diversity values, always in the abdomen and chin. The surgery procedures that had undergone implied shaving the abdomen and were followed by oral medication administration (sometimes antibiotics), which could be associated with the lower alpha diversity values. Larger studies should be performed to corroborate this observation, since the finding was based upon only 4 dogs.

When comparing dogs from the US and European cohorts, the expected result would be a tight cluster for the samples from the homogeneous US cohort and a diffuse if any clustering for dogs from the European cohort. In contrast to the well-controlled US cohort (same crossbred dogs, similar ages, same diet, shared environment, samples obtained and processed as a batch, etc.), dogs from the European cohort were collected along 18 months and were pet dogs from different households that did not interact with each other, with different ages and genetic background. Even considering this heterogeneity, the European dogs clustered together in a single group differing from the environmental well-controlled US cohort that presented a more spread cluster (Additional file 12). These clustering could be associated to the geographic region and its associated environment, as it has already been described for humans with geography [22, 27], geographical isolation [26], or urbanization [20, 28, 29] grouping differently skin microbial communities. However, we cannot discard that this clustering is reflecting variability associated to the laboratory where the samples were extracted. To minimize the variability at technical level between both cohorts, one person was present in both studies (AC), the samples were obtained and extracted with the same protocol, PCRs were performed by the same person in the same laboratory (AC), and sequencing was performed in the same facilities using the same sequencer.

Finally, we should note two main limitations of this study. Despite detecting a clear environmental effect with bacteria from the environment in some skin sites, we did not have environmental samples to strongly support these findings. Future studies of skin microbiota should consider sampling not only the animals, but also their environment even if they are sharing it. Moreover, regarding temporality, we could not distinguish between the season of birth and the time spent in the kennel, since these two variables completely overlap. Longitudinal studies on dogs living together could give some insights on this hypothesis.

Understanding the skin microbiota of the healthy skin will allow a better knowledge of the intrinsic variability in health and the assessment of what is an altered state. It will also provide a background to develop its clinical applications [31] such as identifying an altered skin microbiota landscape or developing personalized therapies aimed at shifting the balance toward a healthy skin microbiota, promoting beneficial bacteria growth rather than killing all bacteria. Thus, to overcome the individual variability inherent to skin microbiota studies, we would recommend longitudinal studies assessing divergences between health and disease comparing affected vs unaffected regions within an individual through time, or, alternatively, the cohort should be large enough and well controlled if case-control studies are preferred.

## Conclusions

The individual drives the skin microbiota variability in healthy dogs, followed by the skin site. Environmentally associated bacteria could be reflecting the different degrees of exposure of each skin site and each dog. Network analyses elucidated bacterial interactions within each skin site and between skin sites for the chin, abdomen, axilla, and the perianal region. When analyzing each skin site independently to assess host-specific factors, we found that temporality (season of birth and time spent in the kennel) affected all the skin sites and specially the inner pinna. We also found taxonomic differences among male and female dogs on the abdomen, axilla, and back.

## Additional files

Additional file 1: Pedigree chart of the dogs included in this study. Circles represent female, and rectangles represent male. In blue are dogs born from January to May that had spent at least 5.5 months in the kennel (T1 group), and in red are dogs born from June to September that had spent 2.5 months in the kennel (T2 group). (DOCX 275 kb)

Additional file 2: Information about the dogs included in the study. Samples were collected April 27, 2016. (XLSX 15 kb)

Additional file 3: OTU tables at genus level. A) OTU table at genus level including all the samples. B) OTU table at genus level collapsed by individual. C) OTU table at genus level collapsed by site. (XLSX 1070 kb)

Additional file 4: Taxonomic composition per sample included at phylum level. (DOCX 416 kb)

Additional file 5: Alpha diversity values and statistics. A) Alpha diversity values for all samples. B) Differences on alpha diversity values among groups regarding different variables. C) Alpha diversity values of dogs that had undergone surgery prior to sampling. (XLSX 31 kb)

Additional file 6: ANOSIM *R* values for each pair of skin sites and both for weighted and unweighted UniFrac matrices. (XLSX 10 kb)

Additional file 7: Differentially distributed families based on skin site. Histogram of linear discriminant analysis (LDA) effect size (LefSe) up to family level for differentially abundant distributed taxa (*α* = 0.05, LDA score > 3). (DOCX 548 kb)

Additional file 8: Network output. CoNet output tables with edge and node information. (XLSX 431 kb)

Additional file 9: Taxonomies of the highly connected nodes obtained through BLAST. (XLSX 10 kb)

Additional file 10: Unweighted UniFrac beta diversity PCoA plot per skin site colored by temporality. In blue are dogs born from January to May that had spent at least  5.5 months in the kennel (T1 group), and in red are dogs born from June to September that had spent 2.5 months in the kennel (T2 group). (DOCX 846 kb)

Additional file 11: Differentially distributed families based on temporality. Histogram of linear discriminant analysis (LDA) effect size (LEfSe) for differentially abundance distribution (*α* = 0.05, LDA score > 3). (DOCX 451 kb)

Additional file 12: Geographical origin effect on beta diversity for back and abdomen samples. Samples from this study (USA) were merged with previous samples (Spain) [9] as well as two other unpublished individuals. Unweighted UniFrac beta diversity plots of (A) dorsal back and (B) abdomen samples colored by geographical origin with their associated ANOSIM and adonis values. (DOCX 191 kb)
