# Supplementary material for: Individual signatures and environmental factors shape skin microbiota in healthy dogs
Source: Microbiome. 2017 Oct 13;5:139. doi: 10.1186/s40168-017-0355-6 (PMC5640918; doi:10.1186/s40168-017-0355-6)

**Additional File 1. Pedigree chart of the dogs included in this study.** Circles represent female and rectangles represent male. In blue, dogs born from January to May that had spent at least 5.5 months in the kennel (T1 group) and in red dogs born from June to September that had spent 2.5 months in the kennel (T2 group).


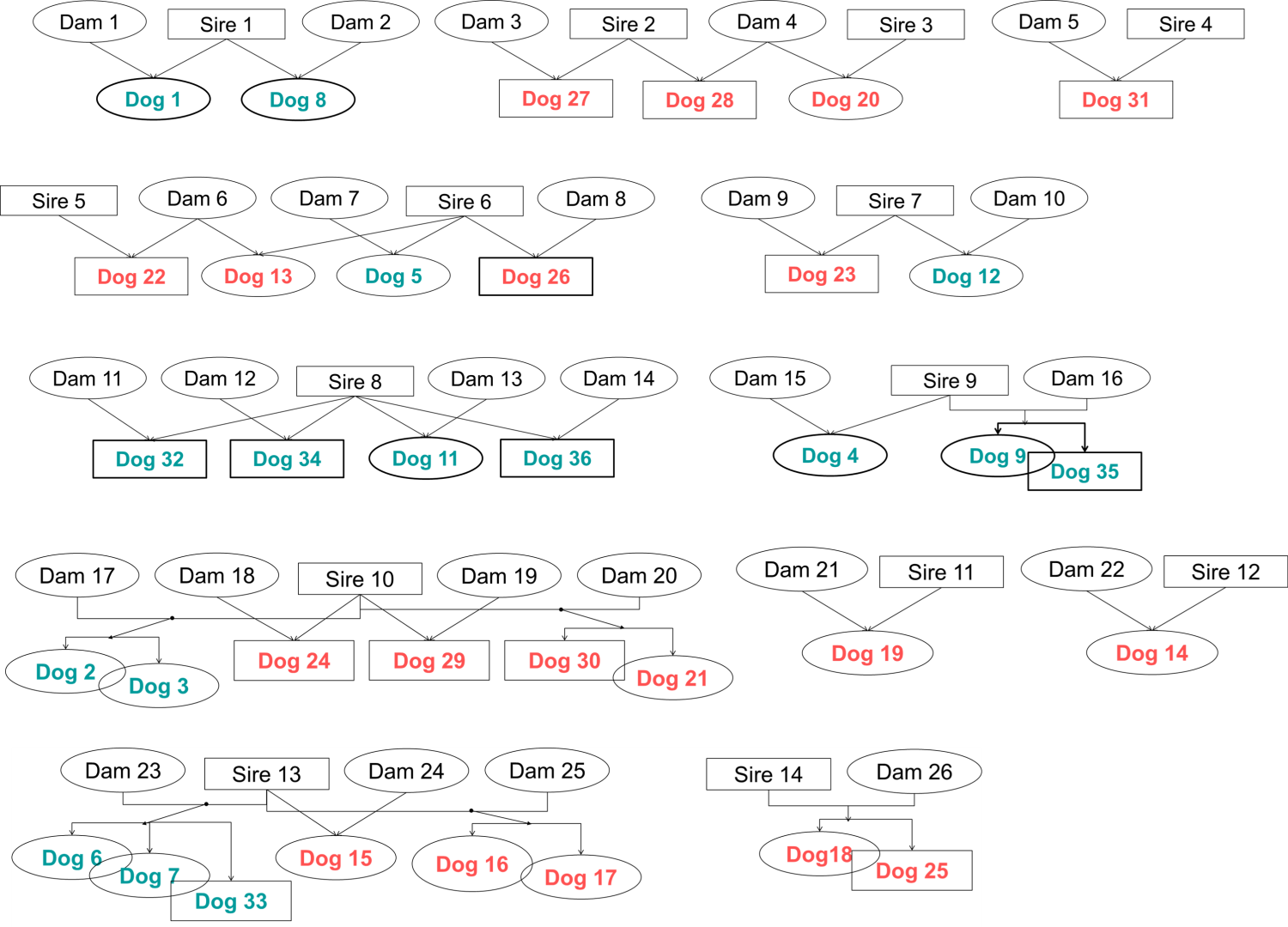

Supplement: Supplementary file 1 — Pedigree chart of the dogs included in this study. Circles represent female, and rectangles represent male. In blue are dogs born from January to May that had spent at least 5.5 months in the kennel (T1 group), and in red are dogs born from June to September that had spent 2.5 months in the kennel (T2 group). (DOCX 275 kb) [file 40168_2017_355_MOESM1_ESM.docx]
