# Supplementary figures and images for: Individual signatures and environmental factors shape skin microbiota in healthy dogs
Source: Microbiome. 2017 Oct 13;5:139. doi: 10.1186/s40168-017-0355-6 (PMC5640918; doi:10.1186/s40168-017-0355-6)

**Additional File 4.** Taxonomic composition per sample included at phylum level.


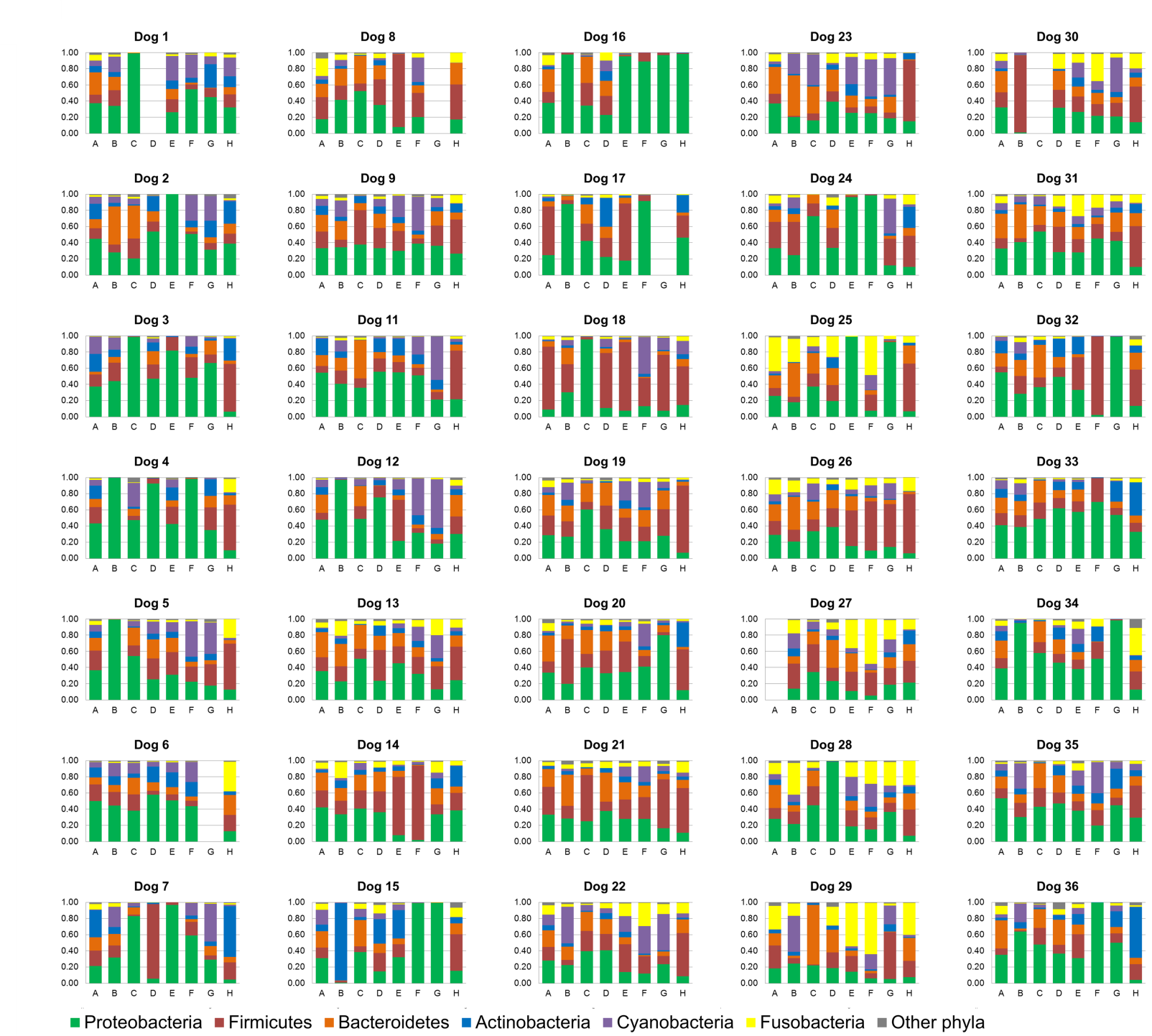

Supplement: Supplementary file 4 — Taxonomic composition per sample included at phylum level. (DOCX 416 kb) [file 40168_2017_355_MOESM4_ESM.docx]
