# Supplementary material for: Individual signatures and environmental factors shape skin microbiota in healthy dogs
Source: Microbiome. 2017 Oct 13;5:139. doi: 10.1186/s40168-017-0355-6 (PMC5640918; doi:10.1186/s40168-017-0355-6)
Supplement: Supplementary file 7 — Differentially distributed families based on skin site. Histogram of linear discriminant analysis (LDA) effect size (LefSe) up to family level for differentially abundant distributed taxa (α = 0.05, LDA score > 3). (DOCX 548 kb) [file 40168_2017_355_MOESM7_ESM.docx]

**Additional File 7.** **Differentially distributed families based on skin site.** Histogram of linear discriminant analysis (LDA) effect size (LEfSe) up to family level for differentially abundant distributed taxa (α = 0.05, LDA score >3).

**
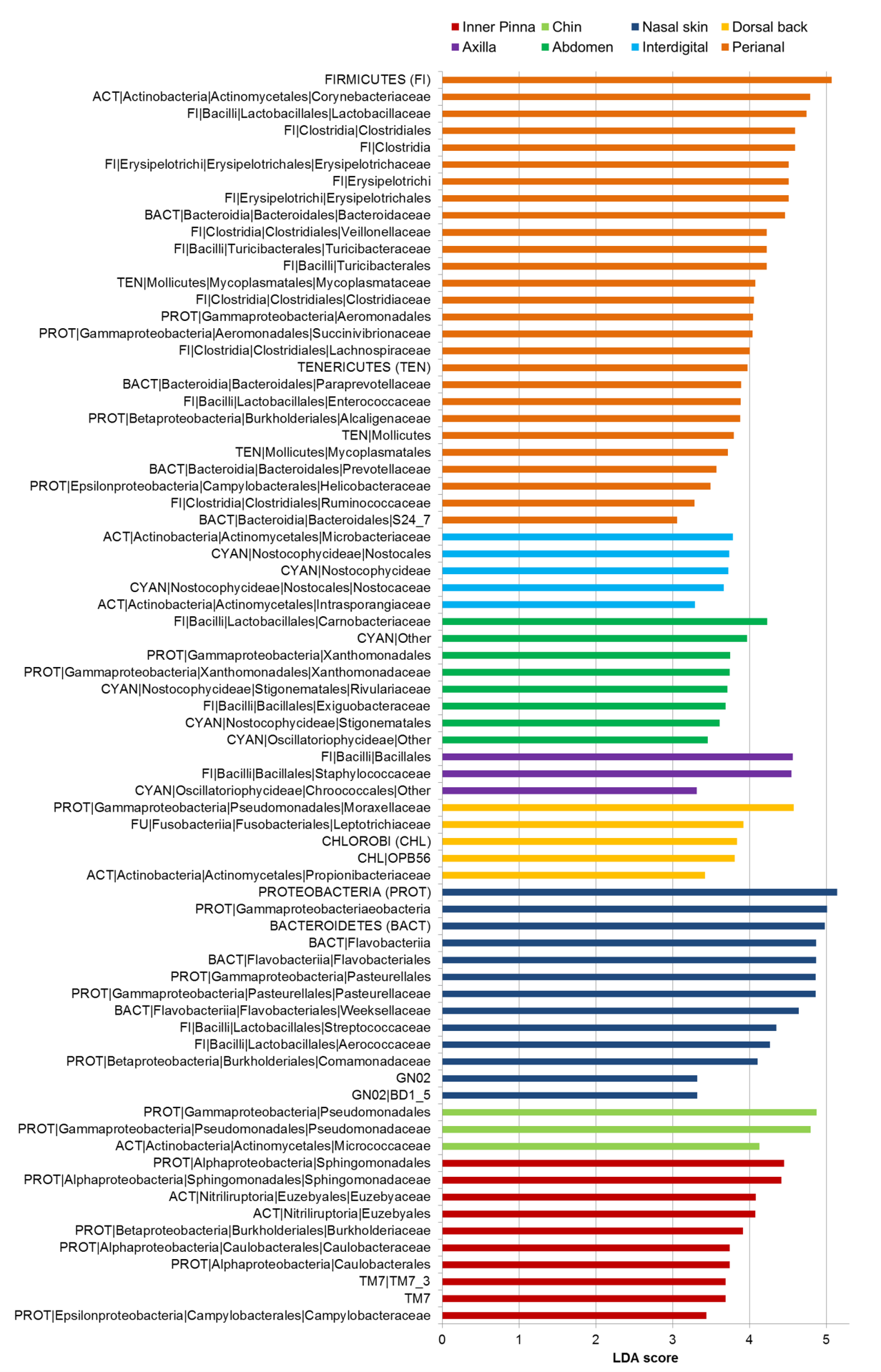
**
