# Supplementary material for: Individual signatures and environmental factors shape skin microbiota in healthy dogs
Source: Microbiome. 2017 Oct 13;5:139. doi: 10.1186/s40168-017-0355-6 (PMC5640918; doi:10.1186/s40168-017-0355-6)

**Additional File 10. Unweighted UniFrac beta diversity PCoA plot per skin site colored by temporality.** In blue, dogs born from January to May that had spent at least 5.5 months in the kennel (T1 group) and in red, dogs born from June to September that had spent 2.5 months in the kennel (T2 group).

(A) Inner pinna (B) Chin


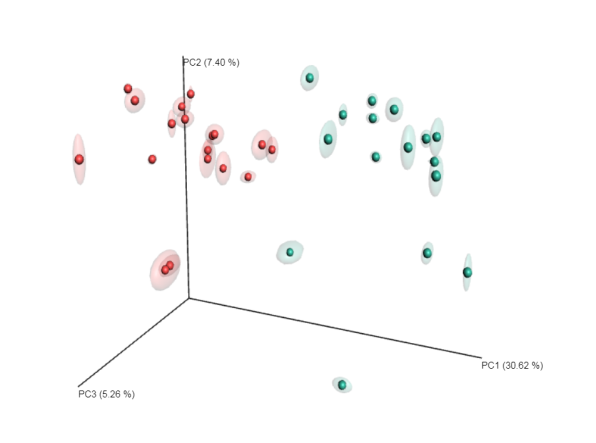

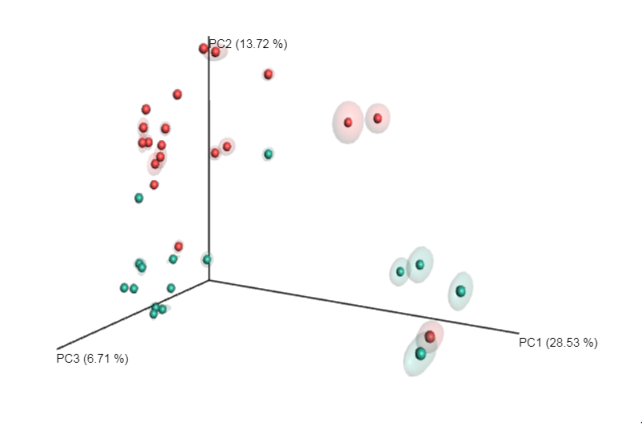


(C) Nasal skin (D) Dorsal back


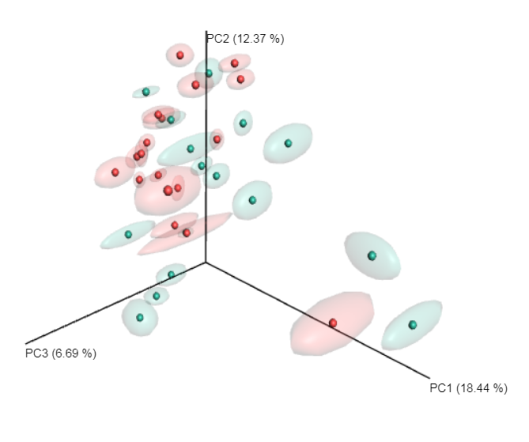

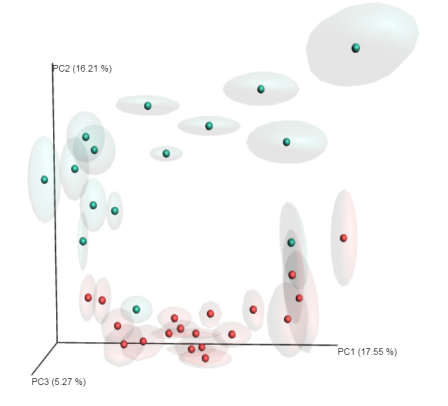


(E) Axilla (F) Abdomen


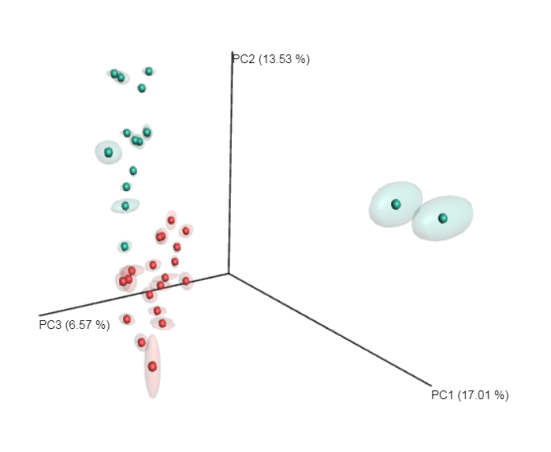

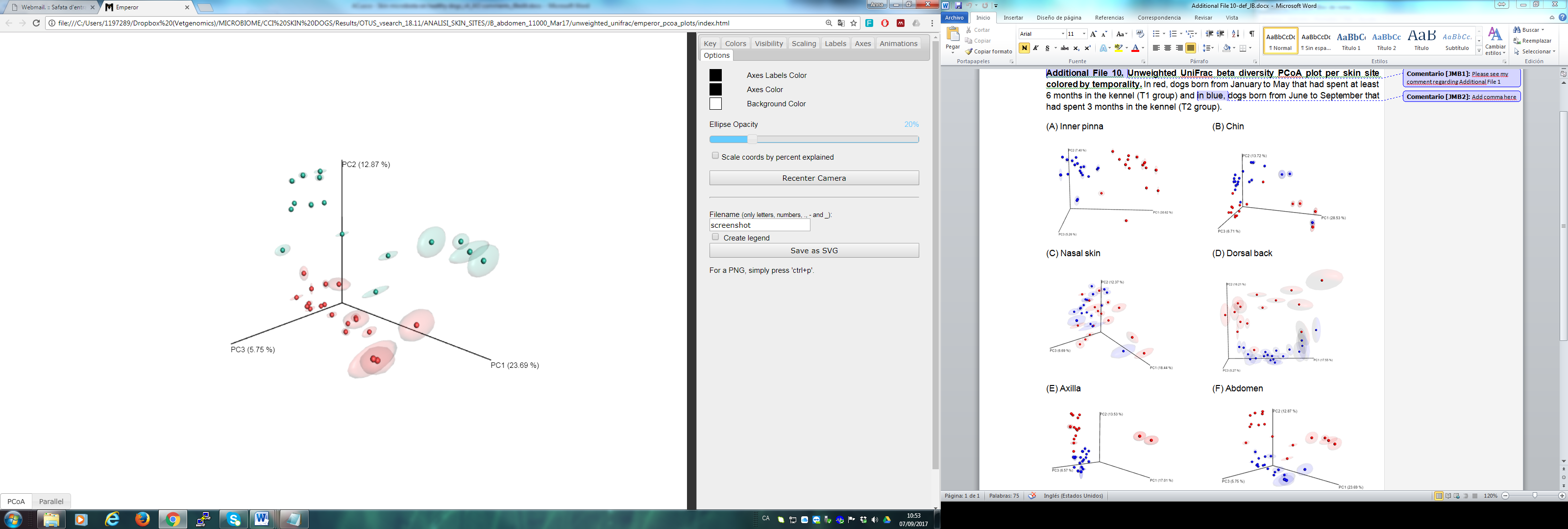


(G) Interdigital region (H) Perianal area


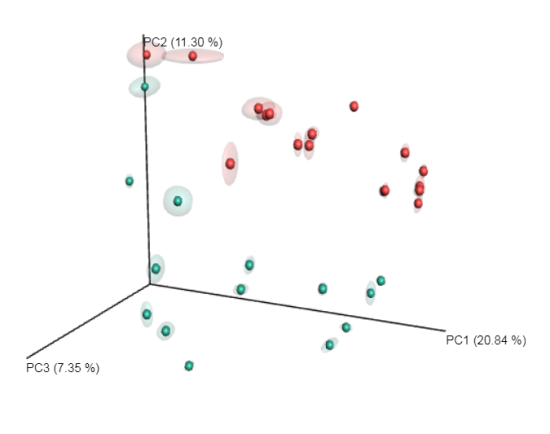

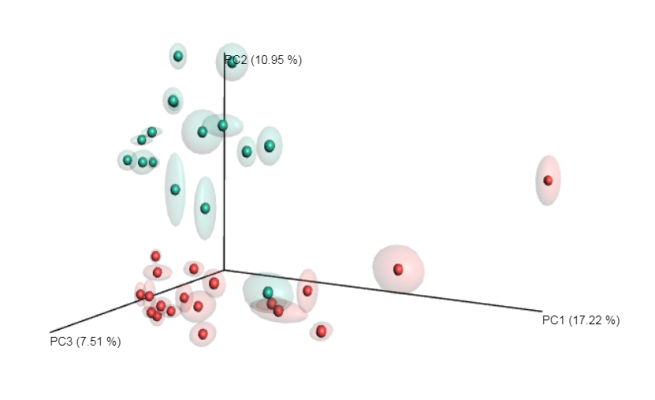

Supplement: Supplementary file 10 — Unweighted UniFrac beta diversity PCoA plot per skin site colored by temporality. In blue are dogs born from January to May that had spent at least 5.5 months in the kennel (T1 group), and in red are dogs born from June to September that had spent 2.5 months in the kennel (T2 group). (DOCX 846 kb) [file 40168_2017_355_MOESM10_ESM.docx]
