# Supplementary material for: Individual signatures and environmental factors shape skin microbiota in healthy dogs
Source: Microbiome. 2017 Oct 13;5:139. doi: 10.1186/s40168-017-0355-6 (PMC5640918; doi:10.1186/s40168-017-0355-6)
Supplement: Supplementary file 11 — Differentially distributed families based on temporality. Histogram of linear discriminant analysis (LDA) effect size (LEfSe) for differentially abundance distribution (α = 0.05, LDA score > 3). (DOCX 451 kb) [file 40168_2017_355_MOESM11_ESM.docx]

**
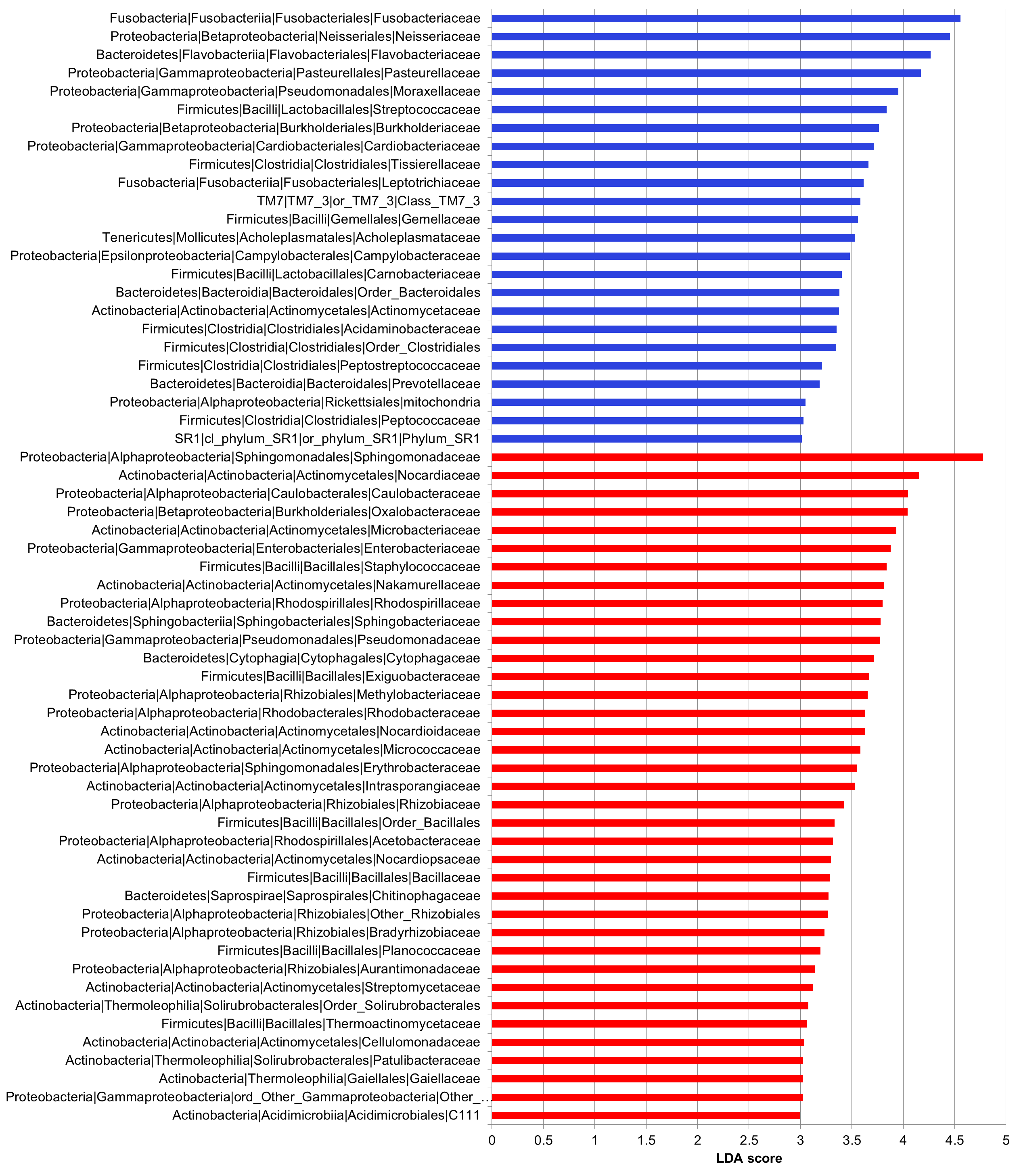
Additional File 11. Differentially distributed families based on Temporality.** Histogram of linear discriminant analysis (LDA) effect size (LEfSe) for differentially abundance distribution (α = 0.05, LDA score >3).
