# Supplementary material for: Individual signatures and environmental factors shape skin microbiota in healthy dogs
Source: Microbiome. 2017 Oct 13;5:139. doi: 10.1186/s40168-017-0355-6 (PMC5640918; doi:10.1186/s40168-017-0355-6)

**Additional File 12. Geographical origin effect on beta diversity for back and abdomen samples.** Samples from this study (USA) were merged with previous samples (Spain) [ 9 ] as well as two other unpublished individuals. Unweighted UniFrac beta diversity plots of (A) dorsal back and (B) abdomen samples colored by geographical origin with their associated ANOSIM and adonis values.


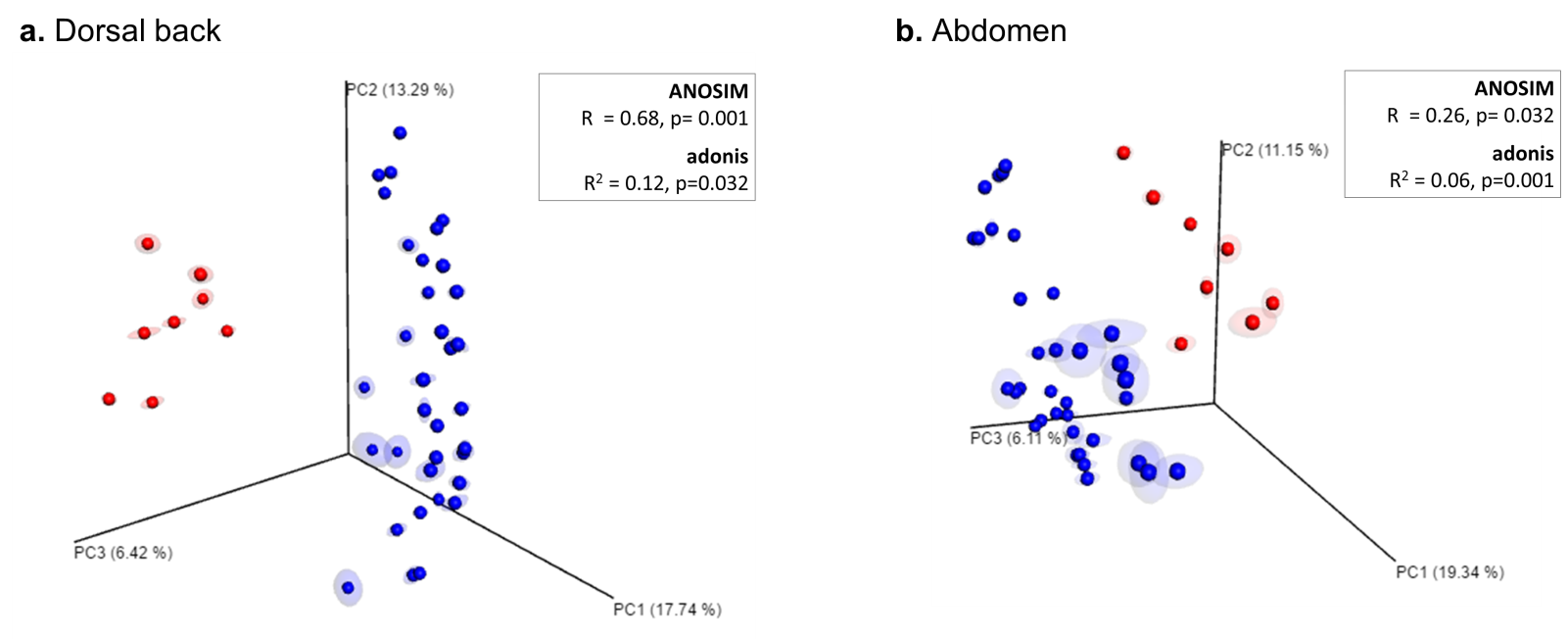

Supplement: Supplementary file 12 — Geographical origin effect on beta diversity for back and abdomen samples. Samples from this study (USA) were merged with previous samples (Spain) [9] as well as two other unpublished individuals. Unweighted UniFrac beta diversity plots of (A) dorsal back and (B) abdomen samples colored by geographical origin with their associated ANOSIM and adonis values. (DOCX 191 kb) [file 40168_2017_355_MOESM12_ESM.docx]
